# Comparison of a bronchoscopic microsample probe with bronchoalveolar lavage to measure cytokine levels in critically ill patients

**DOI:** 10.1186/cc10679

**Published:** 2012-03-20

**Authors:** V Grover, LE Christie, P Charles, P Kelleher, P Shah, S Singh

**Affiliations:** 1 Chelsea and Westminster Hospital, London, UK

## Introduction

The use of bronchoalveolar lavage (BAL) to investigate inflammatory lung disease in the critically ill may not be tolerated in hypoxic patients. Furthermore, soluble protein analysis of BAL fluid suffers from inaccuracies related to saline dilution. The bronchoscopic microsample (BMS) probe allows absolute cytokine levels in epithelial lining fluid (ELF) to be measured directly without lavage [1]. We compared cytokine levels from ELF obtained by the BMS probe with those from BAL, to verify its utility in critical illness.

## Methods

We recruited 45 patients into five groups in whom BMS and BAL were conducted sequentially: two ventilated with ALI/ARDS, six with burns inhalational injury (five ventilated), 15 with COPD, 18 with interstitial lung disease and four healthy patients. The BMS probe was bronchoscopically inserted to the subsegmental level in order to contact the mucosa for 5 to 7 seconds, collecting approximately 20 μl ELF [1]. BAL was performed with 150 ml of 0.9% saline, discarding the first 20 ml (bronchiolar fraction). We assayed IL-1, IL-6, IL-8, TNFα and G-CSF. Comparisons between paired cytokine ELF concentrations in BMS and BAL were analysed using the nonparametric Wilcoxon's test and Spearman's correlation coefficient.

## Results

The critically ill patients were aged 18 to 84 years (APACHE II 12 to 21). One patient had ARDS due to urinary tract infection and another related to pneumonia. No adverse incidents noted were noted. Overall, cytokine levels were all higher in the BMS group than BAL (*P *< 0.0001), consistent with ELF dilution by saline lavage. The ratio of BMS-derived cytokine to BAL for each patient group did not differ significantly. Spearman coefficients (*r*) for IL-1, IL-6, IL-8, TNFα and G-CSF were 0.38, 0.52, 0.25, 0.38 and 0.40. All correlations were significant (*P *< 0.01) except for IL-8 (*P *= 0.05). Both sampling methods demonstrated a gradation of cytokine level, with burns and ALI/ARDS having significantly higher levels than patients with stable chronic lung disease or healthy controls.

## Conclusion

The BMS probe was well tolerated and provided cytokine data comparable to that obtained by BAL in acute and chronic respiratory diseases. The BMS probe may have utility as a biomarker sampling modality in patients where clinicians have concerns over conventional BAL.
